# Partial sequencing of the bottle gourd genome reveals markers useful for phylogenetic analysis and breeding

**DOI:** 10.1186/1471-2164-12-467

**Published:** 2011-09-27

**Authors:** Pei Xu, Xiaohua Wu, Jie Luo, Baogen Wang, Yonghua Liu, Jeffrey D Ehlers, Sha Wang, Zhongfu Lu, Guojing Li

**Affiliations:** 1 Institute of Vegetables, Zhejiang Academy of Agricultural Sciences, Hangzhou 310021, PR China; 2 Institute of Digital Agricultural Research, Zhejiang Academy of Agricultural Sciences, Hangzhou 310021, PR China; 3 Department of Botany and Plant Sciences, University of California, Riverside, CA 92521-0124 USA

## Abstract

**Background:**

Bottle gourd [*Lagenaria siceraria *(Mol.) Standl.] is an important cucurbit crop worldwide. Archaeological research indicates that bottle gourd was domesticated more than 10,000 years ago, making it one of the earliest plants cultivated by man. In spite of its widespread importance and long history of cultivation almost nothing has been known about the genome of this species thus far.

**Results:**

We report here the partial sequencing of bottle gourd genome using the 454 GS-FLX Titanium sequencing platform. A total of 150,253 sequence reads, which were assembled into 3,994 contigs and 82,522 singletons were generated. The total length of the non-redundant singletons/assemblies is 32 Mb, theoretically covering ~ 10% of the bottle gourd genome. Functional annotation of the sequences revealed a broad range of functional types, covering all the three top-level ontologies. Comparison of the gene sequences between bottle gourd and the model cucurbit cucumber (*Cucumis sativus*) revealed a 90% sequence similarity on average. Using the sequence information, 4395 microsatellite-containing sequences were identified and 400 SSR markers were developed, of which 94% amplified bands of anticipated sizes. Transferability of these markers to four other cucurbit species showed obvious decline with increasing phylogenetic distance. From analyzing polymorphisms of a subset of 14 SSR markers assayed on 44 representative China bottle gourd varieties/landraces, a principal coordinates (PCo) analysis output and a UPGMA-based dendrogram were constructed. Bottle gourd accessions tended to group by fruit shape rather than geographic origin, although in certain subclades the lines from the same or close origin did tend to cluster.

**Conclusions:**

This work provides an initial basis for genome characterization, gene isolation and comparative genomics analysis in bottle gourd. The SSR markers developed would facilitate marker assisted breeding schemes for efficient introduction of desired traits.

## Background

Bottle gourd [*Lagenaria siceraria *(Mol.) Standl.] (2n = 2x = 22), also known as calabash or opo squash, is a diploid belonging to the genus *Lagenaria *of the *Cucurbitaceae *family [1]. Phylogenetically, bottle gourd is close to many economically important cucurbit species including cucumber and melon that belong to the genus of *Cucumis*, as well as watermelon that belong to the genus *Citrullus*. Worldwide, bottle gourd is grown for its fruit either being harvested young and used as a vegetable or harvested mature and used as a bottle, utensil, or pipe. The fresh fruit, which usually has a light green smooth skin and a white flesh, is frequently used in many regions of Asia and Africa as either a stir-fry or soup vegetable ingredient [2]. Another recent utilization of bottle gourd is as rootstocks for watermelon against soil-borne diseases and low soil temperature [3,4].

Bottle gourd was one of the first crops to be domesticated. Based on archaeological evidence, bottle gourd is presumed to have been domesticated in Africa [5,6], and might have dispersed to the New World by ocean currents or by human migration in pre-historic times [7,8]. Africa is believed to be the centre of genetic diversity for bottle gourd, although wild progenitors of bottle gourd have not been identified there [6]. Substantial morphological variation for fruit and seed size, shape, color and rind hardness exists in the bottle gourd gene pool [8-10]. Yetisir et al. observed a wide range of morphological variation among Turkish bottle gourd accessions despite the fact that this region is not a center of origin of the crop [11].

At present, very few molecular genetic/genomic resources are publically available for bottle gourd. Achigan-Dako et al. measured the genome size of bottle gourd and showed that the nuclear 2C-value of DNA was around 0.734 pg, which is estimated to be equal to ~ 334 Mb [12]. In spite of the relatively small genome size of bottle gourd, there are only dozens of bottle gourd DNA sequences available in the public DNA database, making it unfeasible to identify bottle gourd genes or to analyze their functions. A limited number of anonymous random amplified polymorphic DNA (RAPD) markers have been described [10,13], but there has been no locus specific DNA markers such as microsatellite (SSR), sequence tagged site (STS) or single nucleotide polymorphism (SNP) markers available for bottle gourd so far. Also unclear is the extent of genome conservation/diversification between bottle gourd and other important cucurbit species such as the model cucumber (*Cucumis sativus *L.), which serves as the basis for comparative genomic analysis across cucurbit species.

Microsatellites, or simple sequence repeats (SSRs), are short repeat motifs usually associated with a high level of frequency of length polymorphism. With the advantages of being stable, PCR-based and relatively low-cost, SSR markers are one of the best choices for genetic research and molecular breeding. SSR markers can be developed, in case of the availability of large number of DNA sequences, *in silico *[14], or experimentally [15]. Traditionally, the experimental approach requires the construction of a genomic library enriched for repeated motifs, hybridization and isolation of microsatellite containing clones, sequencing of positive clones and primer design [16]. Most of these steps, especially the hybridization/isolation step, are expensive and time-consuming. Recent emerging 'next generation' sequencing technique, for instance, the 454 Genome Sequencer FLX (GS-FLX Titanium) shotgun System (Roche, Penzberg, Germany), provides a powerful alternative for generating a tremendous number of DNA sequences for genomics study and marker development. Instead of creating a conventional genomic library enriched for microsatellites, GS-FLX Titanium system sequences a shotgun library in a high-throughput manner, producing tens of thousands of reads around 300-400 bp. By mining the sequence reads, SSR-containing sequences can be identified. Using this technology, we partially sequenced the bottle gourd genome. Through assembling and annotating the sequence reads, tens of thousands of genes with broad range of functional types were recognized. Moreover, hundreds of microsatellite markers were developed using the sequencing data, which are invaluable in future marker assisted breeding and phylogeny analysis. The markers were then applied to a range of bottle gourd accessions to assess genetic diversity to enable more efficient parental line selection for breeding purposes and to dissect the genetic factors underlying morphological variations.

## Methods

### Plant materials

**Figure 1:**
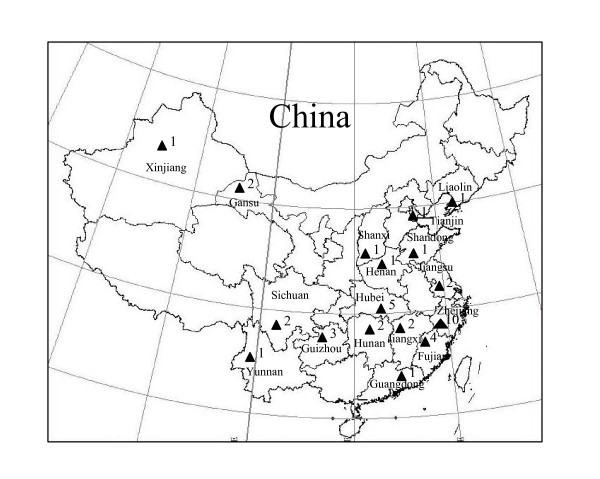
**Distribution of 44 Chinese bottle gourd accessions used in the current study**. The solid triangles indicate collection sites of the materials, and the number following each triangle indicate number of accessions collected from the site.

Forty-four accessions representing geographically and phenotypically different bottle gourd germplasm in China were used in this study (Figure 1; Table 1). The bottle gourd accession used for GS-FLX Titanium sequencing is 'Hangzhou gourd', a landrace from southern China. One accession of each of the following four cucurbits i.e. bitter gourd (*Momordica charantia *L.), loofah [*Luffa acutangula *(L.) Roxb], pumpkin *(Cucurbita pepo *L.) and watermelon [*Citrullus lanatus *(Thunb.)] were also used.

**Table 1:** Cultivar or accession, origin, fruit shape class and type of the genotypes assayed for SSR polymorphisms

**No**.	Cultivar or Accession	Origin	Fruit shape	Type
1	Longyan April Gourd	Fujian province	Round	Landrace
2	D3	Fujian province	Pyriform	Landrace
3	Zhangping Qiye Gourd	Fujian province	Pyriform	Landrace
4	D1	Fujian province	Pyriform	Landrace
5	Linxia Gourd	Gansu province	Slender straight	Landrace
6	Wudu Gourd	Gansu province	Slender straight	Landrace
7	Early Gourd	Guangdong province	Slender straight	Landrace
8	Yuan Gourd	Guizhou province	Tubby	Landrace
9	Songtao Gourd	Guizhou province	Pyriform	Landrace
10	Taijiang Gourd	Guizhou province	Tubby	Improved cultivar
11	Xincai Gourd	Henan province	Slender straight	Landrace
12	Nanxiu	Hubei province	Slender straight	Improved cultivar
13	Xiaogan Gourd	Hubei province	Slender straight	Landrace
14	Qingxiu	Hubei province	Slender straight	Landrace
15	Zhushan Gourd	Hubei province	Pyriform	Landrace
16	Hanlong Qingyu	Hubei province	Slender straight	Improved cultivar
17	Round Gourd No. 1	Hunan province	Round	Landrace
18	V103	Hunan province	Slender straight	Landrace
19	White Gourd	Jiangsu province	Tubby	Landrace
20	Duantong	Jiangsu province	Tubby	Landrace
21	Ganxin	Jiangxi province	Slender straight	Improved cultivar
22	Wheat Gourd	Jiangxi province	Slender straight	Landrace
23	Little Gourd	Liaoning province	Tubby	Landrace
24	Qingzhen	Shandong province	Slender straight	Landrace
25	Puxian Gourd	Shanxi province	Slender straight	Landrace
26	Long Gourd J010	Sichuan province	Slender straight	Landrace
27	Little Seeded Gourd	Sichuan province	Slender straight	Landrace
28	Jinsheng	Tianjin province	Tubby	Landrace
29	Long Gourd	Unknown	Slender straight	Landrace
30	G65	Unknown	Slender straight	Landrace
31	G32	Unknown	Pyriform	Landrace
32	Xinxuan	Unknown	Tubby	Landrace
33	J162	Xinjiang province	Slender straight	Landrace
34	Yunnan Gourd	Yunnan province	Pyriform	Landrace
35	Yongzhen No.1	Zhejiang province	Slender straight	Improved cultivar
36	Xiaoshan Garden Gourd	Zhejiang province	Slender straight	Landrace
37	Quanhua Gourd	Zhejiang province	Slender straight	Landrace
38	G63	Zhejiang province	Slender straight	Landrace
39	Xiaoshan Long Gourd	Zhejiang province	Slender straight	Landrace
40	G61	Zhejiang province	Slender straight	Landrace
41	G62	Zhejiang province	Slender straight	Landrace
42	Shaoxing Gourd	Zhejiang province	Slender straight	Landrace
43	Hangzhou Gourd	Zhejiang province	Slender straight	Landrace
44	Anji Gourd	Zhejiang province	Slender straight	Landrace

### DNA extraction

Genomic DNA was extracted from leaves of two-week-old seedlings using a modified CTAB method [17].

### DNA library construction and sequencing

To construct DNA library for GS-FLX Titanium sequencing, 5 mg of genomic DNA were fragmented into 300-800 bp by nebulization. Short adaptors were then ligated to the 3' and 5' ends. Emulsion PCR (emPCR) was carried out at a concentration of 1 copy per bead in six emulsion oils, to give 43,800 enriched beads. Amplified fragments were sequenced on 1/4th of an LR70 plate. The reads from GS-FLX Titanium sequencing were assembled with the software Newbler (http://rcc.uga.edu/software/app/newbler_GS_De_Novo_Assembler/) under default parameters.

### Functional annotation of genes and gene ontology analysis

Functional annotation of the sequences was performed by BLAST × search against the NCBI no-redundant (nr) protein database using the assembled contigs/singletons as queries. The cut-off value for significance was set as e^-10^. A putative gene ontology and functional category were obtained on the basis of GO Consortium (http://www.geneontology.org/) by BLAST2GO (http://www.blast2go.de).

### Alignment of gene sequences between genomes

The cucumber genome sequence was downloaded from Phytozome (ftp://ftp.jgi-psf.org/pub/JGI_data/phytozome/v6.0/Csativus/). A total of 16,135 bottle gourd contigs/singletons, which were functionally annotable under an E-value < e^-10 ^and thus were considered originated from the gene space of the genome, were compared with the cucumber genome sequence by BLAST N under an E-value threshold of e^-10 ^in at least 100 bp overlap. For comparison of the *Cryptochrome 1 *molecular clock marker genes and the UDP-glucosyltransferase genes among species, each gene sequence was download from Genbank (http://www.ncbi.nlm.nih.gov) under the accession numbers of AB073546.1 (*OsCRY1a*, rice), EF601539.1 (*TaCRY1a*, wheat), AB498928.1 (*GmCRY1*, soybean), FE690583.1 (*PvCRY1*, common bean) or from the cucurbit unigene database (http://www.icugi.org/cgi-bin/ICuGI/EST/home.cgi?organism=melon) under the accession number of MU45735 (*CmCRY1*, melon) and MU59780 (UDP-glucosyltransferase gene, melon), with the exception of cucumber *CRY1 *and UDP-glucosyltransferase genes, whose sequences were extracted from the cucumber genome database [18].

### Microsatellites mining, primer design and SSR assay

The assembled contigs/singletons sequences were screened for perfect microsatellites using the software mreps 2.5 (http://bioinfo.lifl.fr/mreps/) [19]. The software Websat (http://wsmartins.net/websat/) was used to design primers flanking SSRs [20]. Only sequences containing SSRs equal to or longer than 20 bp were used for primer design. The procedure of SSR assay followed Xu et al. [14].

### Analysis of genetic diversity

The alleles present in each genotype were scored visually for each SSR locus. Number of alleles and allele frequency per locus were calculated manually. The computer program PIC_Calc 0.6 (http://www.esnips.com/doc/9171097b-ac41-424a-9d35-e7d4e540ec9f/Picalc) was used to measure the polymorphism information content (PIC) value for each SSR locus under the formula PIC = 1-ΣP_ij_^2^, where P_ij _is the frequency of *j*th allele of the *i*th locus [21]. Calculation of Nei's genetic distance (*D*_A_) and principal coordinates analysis (PCoA) were performed with NTSYSpc 2.10 [22]. A dendrogram showing relatedness among the 44 bottle gourd accessions were constructed using the unweighted pair-group method (UPGMA) based on the information of *D*_A_.

## Results

### Summary of the GS-FLX sequencing data

A ¼ run on the GS-FLX system generated 150,253 reads that passed the quality filters, giving a total length of 56,368,975 bp. The length of individual reads ranged from 23 bp to 700 bp, with an average of 375.2 bp. The majority of the read lengths fell between 350 bp and 500 bp. These sequences then were assembled into contigs based on sequence overlaps. After removing 75 long contigs (> 2 kb) that were found from a chloroplast/mitochondrial origin, 3,994 contigs ranging from 100 bp to 1,873 bp with an average length of 1236 bp and 82,522 singletons ranging from 23 bp to 649 bp with an average length of 362 bp were obtained (Table 2). These non-redundant contigs and singletons taken together represent ~32 Mb of the nuclear DNA sequence, covering ~10% of the bottle gourd genome. The original sequencing data is accessible at the DDBJ database under the accession number of DRR001005. The assembled contigs/singletons sequences can be downloaded from ftp://60.191.1.9 under the user name of 'gourd' and password of 'sequence2011'.

**Table 2:** Length distribution of contigs and singletons

Singletons			Contigs		
**Range of length**	**Counts**	**Percentage (%)**	**Range of length**	**Counts**	**Percentage** **(%)**

< 200 bp	15,985	19	< 400 bp	907	22
200-299 bp	7860	9	400-699 bp	2428	60
300-399 bp	13295	16	700-999 bp	480	12
400-499 bp	32331	39	1000-1499 bp	157	3
≥ 500 bp	13051	15	≥ 1500 bp	22	0.6

Overall	82522	100	Overall	3994	100

### Functional annotation of the sequences

BLAST × search against the NCBI GenBank peptide database performed using the 3,994 sequence assemblies and 82,522 singletons resulted in 18,033 annotated sequences under an E-value threshold of e^-10^. After removing putative plastid/mitochondrial sequences and retrotransposon/transposon elements, 16,135 'clean' sequences were maintained. The lengths of the annotated sequences varied from 105 bp to 1,873 bp. Of these, 11,216 (69%) hit a gene function as hypothetical/predicted proteins or unknown/unnamed proteins while 4,919 (31%) sequences had known putative gene function annotations (Additional file 1).

**Figure 2:**
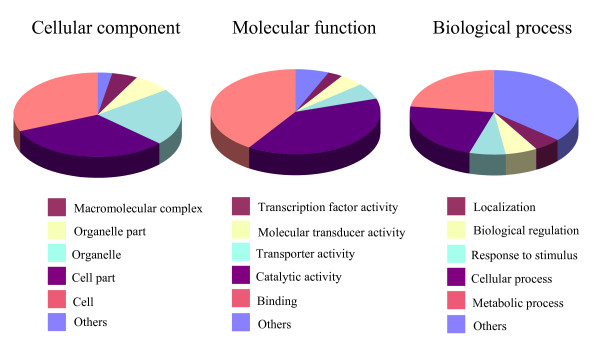
**Gene ontology (GO) categories of 4919 non-redundant contigs/singletons with a putative functional annotation**.

Functional assignments for the 4,919 sequences with putative gene function annotations covered all three top-level ontologies i.e. cellular component, biological process and molecular function. Among those sequences that fell into the functional classification of molecular function, the largest categories were binding (40.8%), followed by catalytic activity (39.1%). In the class of biological process, cellular processing formed the major category (22.5%). Cell part (31.4%) is the dominant group of the cellular component classification (Figure 2).

### Conservation of gene sequences between bottle gourd and cucumber

To estimate the extent of sequence conservation between the gene spaces of bottle gourd and the model cucurbit cucumber, we compared 16,135 bottle gourd contigs/singletons that were assigned a functional annotation with the newly available cucumber genome sequence. BLAST N result showed that 13,370 bottle gourd sequences matched the cucumber genome in at least 100 bp overlap (Additional file 2). As expected, most of the matched sequences occur in the exon regions, giving an average sequence identity value of as high as 90.3%. Six hundred and fourteen bottle gourd sequences (4.6%) had more than 95% identity with cucumber, while 1252 sequences (9.4%) showed relatively low sequence conservation (less than 85% identity). Notably, we found that the gene *Cryprochrome 1 *(*CRY1*), which encodes a blue light receptor ubiquitous throughout the plant kingdom and that is frequently used phylogenic molecular clock marker [23,24], showed an identity value of as high as 93.5% in the conserved C-terminus DAS domains between the two species, demonstrating that the two species are phylogenetically very close. Another conserved plant gene, the UDP-glucosyltransferase gene, showed 85% sequence identity between bottle gourd and cucumber and a much higher sequence identity between melon and cucumber (93%, see discussion below).

### Characterization of microsatellites in bottle gourd

**Figure 3:**
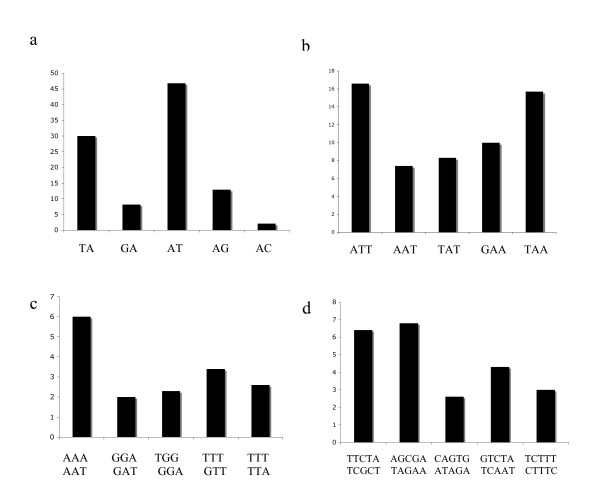
**Frequency of Di- (a), Tri- (b), Hexa- (c) and Deka- (d) nucleotide SSR motifs**. In each panel only the five most frequent nucleotride motifs are shown.

A search against the sequenced bottle gourd genome for microsatellite-containing sequences hit 201 positive contigs and 3815 singletons at the threshold of SSR length ≥ 20 bp, harboring a total of 4395 discrete microsatellites. Of these, dinucleotide and dekanucleotide repeats are the most abundant, each accounting for ~13% of the total number. Trinucleotide repeats is also abundant, while mononucleotide and pentanucleotide repeats are relatively rare (Table 3). The length of the majority of the SSRs ranged from 20 to 56 nucleotides, with the longest up to 244 nucleotides. The number of repeat units varied between 2 and 122. Of the dominant dinucleotide and dekanucleotide repeats, AT/AT and TTCTCTCTCT/AGAGAGAGAA are the most frequent types of motif. AAT/ATT, TTTA/TAAA and AAAAAT/ATTTTT are the most common tri-, tetra- and hexa- nucleotide repeats, respectively (Figure 3). Clearly, AT rich repeats take up the majority of the microsatellites longer than 20 bp in the bottle gourd genome.

**Table 3:** Summary of the presence and type of simple sequence repeats (SSRs) longer than 20 bp

Motif	Number	Percentage (%)	Range of copy number
Mono	69	1.6	20-35
Di	583	13.3	10-122
Tri	471	10.7	7-19
Tetra	190	4.3	5-10
Penta	182	4.1	4-8
Hexa	348	7.9	3-7
Hepta	305	6.9	3-17
Octa	235	5.3	3-6
Ennea	311	7.1	2-14
Deka	585	13.3	2-3
> Deka	1116	25.4	/
Total	4395	100	/

Around 32% of the non-redundant microsatellite-containing sequences were suitable for design of flanking PCR primers. The rest of the microsatellite-containing sequences were less useful in primer development because the microsatellites were too close to fragment ends to enable design of flanking PCR primers. We designed 400 SSR markers (Additional file 3) from the contigs/singletons sequences and tested the amplification of 200 (LSR001-LSR200) of them. Ninety-four percent of the PCR primers amplified products with anticipated sizes (data not shown), demonstrating a high fidelity and efficiency for large scale SSR marker development by the GS-FLX sequencing approach.

### Transferability of the microsatellite markers across species

To test the usefulness of the newly developed microsatellite markers in other understudied cucurbit species, we investigated their transferability to four other cucurbits, i.e. bitter gourd (*Momordica charantia *L.), loofah [*Luffa acutangula *(L.) Roxb], pumpkin *(Cucurbita pepo *L.) and watermelon [*Citrullus lanatus *(Thunb.)] by amplification with 200 primer pairs (LSR001-LSR200). Relatively low cross-species SSR transferability was observed except that between bottle gourd and watermelon who are both members of the subtribe *Benincasinae*, and, as expected, rate of marker transferability showed significant decline with increasing phylogenetic distance (Table 4). Using genomic sequences from non-expressed regions may partially account for the low marker transferability across species.

**Table 4:** Transferability of the bottle gourd SSR markers to four other cucurbits

Species	No. successful amplification	Transferability (%)
Bitter gourd	8	4
Loofah	38	20
Pumpkin	21	11
Watermelon	76	41
Bottle gourd	188	94

### Genetic diversity of 44 Chinese bottle gourd accessions as assessed by SSR markers

Fourteen primer pairs that detected polymorphisms in at least two of the four selected bottle gourd lines, i.e. 'Long gourd', 'Longyan April gourd', 'Nanxiu' and 'Yongzhen No. 1' (data not shown) were used to genotype 44 entries of Chinese bottle gourd accessions (Table 1). A total of 51 alleles with two to eight alleles per locus were detected among the accessions, providing an average allele number of 3.64 per locus. The overall polymorphism information content (PIC) value varied from 0.11 to 0.72 with an average of 0.4 (Table 5).

**Table 5:** Number of alleles and polymorphism information content of the markers used in genetic diversity analysis

Primer name	Repeat type	No. of alleles	PIC
LSR011	ATT	5	0.579
LSR015	CTT	8	0.715
LSR020	G	3	0.384
LSR030	AT	4	0.227
LSR040	TTC	2	0.112
LSR047	TC	3	0.518
LSR056	CTT	4	0.315
LSR063	ATT	3	0.266
LSR074	TA	6	0.540
LSR077	TC	4	0.401
LSR088	ATA	2	0.369
LSR108	AG	3	0.439
LSR109	GA	2	0.354
LSR112	TTCT	2	0.320

**Figure 4:**
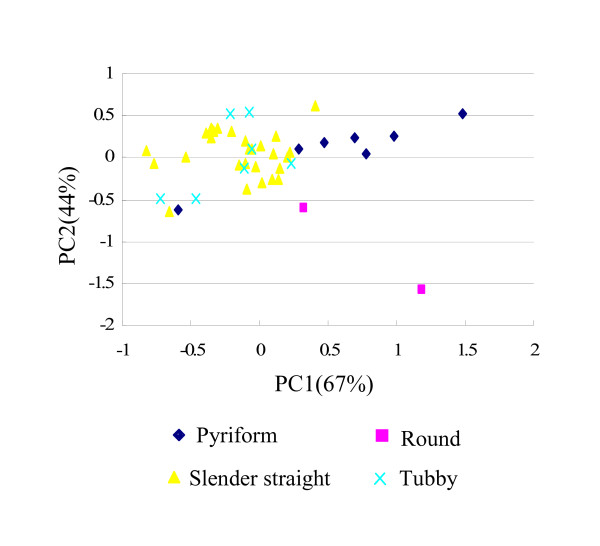
**Two-dimensional principal coordinates analysis of 44 bottle gourd cultivars/accessions**. Different codes of the data points represent fruit shapes of the genotypes.

**Figure 5:**
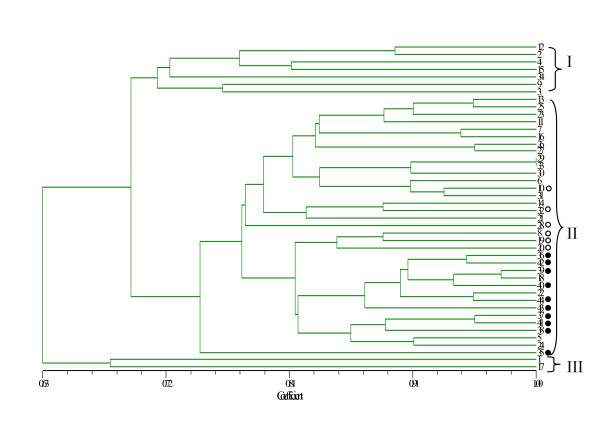
**A dendrogram of the 44 bottle gourd cultivars and accessions constructed using 14 microsatellite polymorphisms**. Numbers indicate cultivar/accession as listed in Table 1. The ten accessions originating from Zhejiang province are marked by solid circles. The six accessions that harbor the intermediate tubby fruit and are clustered together are marked by open circles.

A two-dimensional principal coordinates analysis (PCoA) did not detect significant subgrouping among the 44 lines, while the tendency of certain accessions to congregate together still can be observed (Figure 4). This distribution of the cultivars/landraces in general showed an association with fruit shape rather than geographic origin. For example, accessions with pyriform and tubby fruit formed a cluster in the upper right and upper left corners, respectively, while two round-fruited accessions clustered in the lower right corner. The rest of the accessions exhibited a scattered distribution along the two axes. Consistent with this, the dendrogram constructed from UPGMA analysis showed three major groups, which in general corresponds to the three clusters revealed by PCoA (Figure 5). The smallest group (group III) consisted of the two lines (No. 1 and 17) with round fruits. Lines in Group I were all landraces with a pyriform fruit except for 'Nanxiu' (No. 12), which is a commercial cultivar popular in central China with a slender straight fruit. Group II, the biggest class, consisted of 25 accessions with a slender straight fruit and 7 tubby-fruited accessions with six of the latter showed a clustered distribution in the dendrogram (Figure 5).

Even though no strong association was observed between the subgrouping and geographic origin of these accessions, cultivars or landraces sharing the same or close origins still tend to be clustered together in certain subclades. For instance, all the ten cultivars/landraces from Zhejiang province, a center of cultivation of bottle gourd in China were clustered together in group II (Figure 5).

## Discussion

Through partial sequencing of the genome via the 454 GS-FLX Titanium sequencing platform, we were able to rapidly generate DNA sequence recourses for molecular marker development and genomic inquiry in bottle gourd, an 'orphan crop' for which few genomic resources have been developed thus far. Tens of thousands of sequences with putative functional annotation were identified, which will allow primer design or probe development for gene expression analysis, microarray assay, *in silico *cloning of the genes, as well as comparative analysis among cucurbits. The availability of bottle gourd genome sequences will be helpful to get a better understanding of some bottle gourd or cucurbits specific traits. For example, the sequences information will facilitate the identification of genes responsible for the highly efficient water transport system that is characteristic to bottle gourd and other cucurbits [25], and the hunt for genes related to the bitter taste-causing cucurbitacins biosynthetic pathway in cucurbits [26,27].

We provided the first insight of genome conservation/diversification between bottle gourd and the model cucurbit cucumber. We showed that the extent of gene space conservation between the two species is as high as 90%, demonstrating a close relationship between bottle gourd and cucumber. This is consistent with the result from analyzing the *CRY1 *molecular clock gene, which showed a 93% sequence identity between the two species in the C-terminus DAS domain. This value is higher than that between rice (*Oryza sativa*) and wheat (*Triticum estivum*) (69.4%), two related *Poaceae *species, and even higher than between the warm season legumes soybean (*Glycine max*) and common bean (*Phaseolus vulgaris*) (88%), indicating again that bottle gourd and cucumber are phylogenetically very close. However, the value is lower than that between melon (*Cucumis melo*) and cucumber (95%), which is consistent with the current phylogeny of cucurbits [28]. Similar results were obtained from analyzing the UDP-glucosyltransferase genes, where a much higher level of sequence identity was observed between melon/cucumber (93%) than between bottle gourd/cucumber (82%). Assay of SSR markers transferability across different cucurbit species also supported the known phylogeny and demonstrated that the bottle gourd SSR markers could be selectively used for watermelon (41% amplification rate), and loofah (20% amplification rate) if necessary, due to their relatively higher cross-species transferability.

Another direct use of the sequencing information is to develop large number of microsatellite markers for marker-assisted breeding. The quick generation of over 150,000 sequence entries that enabled development of thousands of SSR markers within only 1 week at low cost is far superior to the traditional, hybridization and Sanger sequencing based method [15,29] in terms of time, labor and other costs. The GS-FLX Titanium system was chosen because it generates longer sequence length (~ 400 bp) per read than most other next generation sequencing systems, which is important for the subsequent design of SSR primers flanking the microsatellite motifs. We identified 4395 SSRs longer than 20 bp from the non-redundant 32 Mb bottle gourd genome sequence, which provides a frequency of 1 SSR per ~7.3 Kb. This frequency is nearly double the estimation from cucumber (1 SSR per ~14.6 Kb) using 3x shotgun genome sequencing data [30], and demonstrates that SSRs could serve as a rich source for marker development in bottle gourd. The high frequency of dinucleotide and trinucleotide repeats is consistent with the situation in most other plant species including the cucurbits cucumber and watermelon [29-31]; however, the significantly high portion of dekanucleotide repeats could be a feature of the bottle gourd genome although dekanucleotide repeats is also common in other plant genomes such as cowpea [31]. The AT-rich nature of the microsatellite motifs is conserved between bottle gourd and cucumber [30].

A dendrogram established based on SSR genotyping of 44 representative China bottle gourd cultivars/landraces didn't detect obvious clustering by geographical location, which is in agreement with Yetisir et al. in which clustering of bottle gourd accessions from Turkey was based around fruit morphology much more than on geographical origin [11]. Founder effects followed by assortive mating, i.e. the original introduction of only limited genetic diversity within fruit types, followed by matings mostly within fruit types, would lead to the patterns of genetic diversity observed. This is supported by the relatively high genetic similarity observed among the bottle gourd lines, which varied between 51.2% and 94.3%. Decker-Walters et al. (2001) characterized 74 landraces/cultivars from a global sample and revealed that the lines from diverse origins (Africa, Asia and the New World) were readily separated [10]. Consistent with the result from Morimoto et al. (2005), fruit shape was found a principal component of the variation and is in general associated with the grouping of the lines based on molecular markers [8]. Our results indicate that China bottle gourd germplasm could be divided into three major groups in terms of fruit shape, i.e. slender straight, tubby and round, although the variation of fruit shape is quantitative. Heiser proposed that bottle gourd plants producing large round fruits are typically native to tropical West Africa, whereas the long, thin, snake-like fruits are considered to be of Asian origin [9]. This, if true, is indicative of a mixed origin of Chinese bottle gourd germplasm. The presence of the pyriform and tubby fruit lines, which are considered an intermediate type, could be indicative of natural or artificial hybridization between the two ancient cultivar groups. Relatively recent human migration events and recent germplasm introduction activities may further blur the patterns of diversity as revealed by the imperfect association between the morphology of the lines and their grouping.

## Conclusions

We report here the generation of 454 GS-FLX Titanium sequencing data of the bottle gourd genome and its application to SSR marker discovery and genetic diversity analysis. The sequence information will allow characterization of the bottle gourd genome, facilitate gene isolation and comparative genomics analysis across species. The SSR markers developed will enable marker assisted breeding of bottle gourd, while the characterization of patterns of diversity among representative China bottle gourd accessions will facilitate the optimal use of genetic resources for breeding. In the near future, with more and more genome sequence information of other cucurbits becoming available [18,32], soon it will be feasible to draw deeper and clearer insights into genome conservation/diversification among related crop cucurbit species.

## Authors' contributions

PX and GL designed the experiments. XW and SW carried out the experiments. BW, YL and ZL participated the field work and trait evaluation. PX analyzed all the data and performed computational analyses, with the assistance from JL. PX drafted the manuscript and JDE and GL revised the manuscript. All authors read and approved the final manuscript.

## Supplementary Material

Additional file 1 **Functional annotation of the contigs/singletons**. The annotation of putative functions of the contigs/singletons with an E-value equal to or smaller than e^-10 ^through BLAST X. Click here for file

Additional file 2 **Sequence comparison between bottle gourd and cucumber**. BLAST N result between bottle gourd gene space sequences and cucumber genome. Highlighted are percentage identity values. Click here for file

Additional file 3 **Bottle gourd SSR markers developed**. Sequences and characteristics of the microsatellite markers developed from bottle gourd contig/singleton sequences. Click here for file
